# ASAXS measurements on ferritin and apoferritin at the bioSAXS beamline P12 (PETRA III, DESY)

**DOI:** 10.1107/S1600576721003034

**Published:** 2021-05-25

**Authors:** D. C. F. Wieland, M. A. Schroer, A. Yu. Gruzinov, C. E. Blanchet, C. M. Jeffries, D. I. Svergun

**Affiliations:** a Institute for Materials Research, Helmholtz-Zentrum Geesthacht, Max-Planck Strasse 1, Geesthacht, 21502, Germany; b European Molecular Biology Laboratory, c/o DESY, Notkestrasse 85, Hamburg, 22607, Germany

**Keywords:** anomalous small-angle X-ray scattering, ASAXS, macromolecules, absorption edges, ions

## Abstract

Small-angle X-ray scattering is widely utilized to study biological macromol­ecules in solution. For samples containing specific (*e.g.* metal) atoms, additional information can be obtained using anomalous scattering. Here, measuring samples at different energies close to the absorption edges of relevant elements provides specific structural details. However, anomalous small-angle X-ray scattering (ASAXS) applications to dilute macromolecular solutions are challenging owing to the overall low anomalous scattering effect. Here, pilot ASAXS experiments from dilute solutions of ferritin and cobalt-loaded apoferritin are reported. These samples were investigated near the resonance X-ray *K* edges of Fe and Co, respectively, at the EMBL P12 bioSAXS beamline at PETRA III, DESY. Thanks to the high brilliance of the P12 beamline, ASAXS experiments are feasible on dilute protein solutions, allowing one to extract the Fe- or Co-specific anomalous dispersion terms from the ASAXS data. The data were subsequently used to determine the spatial distribution of either iron or cobalt atoms incorporated into the ferritin/apoferritin protein cages.

## Abstract 2

Anomalous small-angle X-ray scattering (ASAXS) allows one to obtain information about the spatial distribution of specific atoms in a sample, but its application to dilute biological samples is challenging because of weak anomalous effects. ASAXS experiments from dilute solutions of ferritin and cobalt-loaded apoferritin near the resonance X-ray *K* edges of Fe and Co, respectively, have been performed at the P12 bioSAXS beamline of the EMBL at PETRA III synchrotron, DESY.

## 1. Introduction   

Soft matter and biological systems are actively utilized in applied science and biophysical processes (Henderson *et al.*, 2005 ▸; Holm *et al.*, 2001 ▸). Especially important are the ionic and polar interactions of macromolecules in specific solution environments, which significantly influence structural dynamics and thermodynamic processes (Prabhu, 2005 ▸) and direct interactions between biomolecules and electrolytes (such as metals) for storage, signalling and catalytic purposes. Understanding the incorporation of electrolytes and polyelectrolytes into biological systems is of significant interest at a fundamental level for cellular and structural biology (*Metals in Chemical Biology*, 2008 ▸; Thiele & Gitlin, 2008 ▸; Holm *et al.*, 1996 ▸), and for the development of biomaterials, biosensor catalysts and catalytic environments. One of the key aspects of investigating such systems relates to obtaining information about the spatial distribution of counter-ions around the biopolymers in solution (Ballauff & Jusufi, 2006 ▸).

Answering such scientific questions is not trivial and needs advanced analysis methods. Anomalous small-angle X-ray scattering (ASAXS) is one method that allows one to probe the structure of biopolymers and obtain element-specific information. In an ASAXS experiment, one utilizes the anomalous dispersion of X-rays by specific elements associated with these biopolymers to assess their (time-preserved) spatial distributions. ASAXS is possible at synchrotron X-ray sources because of the ability to fine-tune the X-ray energy, allowing one to perform experiments close to and at an absorption edge of a specific atom. This approach opens up the possibility to directly probe the distribution of bound and associated counter-ions of polymeric bio-systems by selecting the X-ray energy around the edge of a counter-ion of interest (Pabit *et al.*, 2010 ▸; Ballauff & Jusufi, 2006 ▸) and also by performing an element-specific contrast variation (Stuhrmann, 1981*a* ▸, 1985 ▸).

Pioneering ASAXS work on macromolecules was performed as early as 1981, investigating ferritin, haemoglobin and caesium binding around DNA (Stuhrmann, 1981*b* ▸). However, further ASAXS applications mainly involved inorganic samples because of experimental limitations of measurements on biological solutions (Stuhrmann, 1981*a* ▸,*b* ▸, 1985 ▸). Recent progress means that ASAXS on biological samples has become feasible, owing to advances in instrumentation and thanks to reduced background scattering and increased photon flux at modern synchrotron beamlines (Sztucki *et al.*, 2012 ▸, 2010 ▸; Jusufi *et al.*, 2012 ▸). ASAXS has been successfully applied to investigate the ion distributions around polyelectrolytes, DNA and micelles (Ballauff & Jusufi, 2006 ▸; Pabit *et al.*, 2010 ▸; Dingenouts *et al.*, 2004 ▸; Jusufi *et al.*, 2004 ▸). The combination of ASAXS with density functional theory has allowed deeper insights and validation of structural models on the counter-ion distribution and sub-molecular features (Krishnamoorthy *et al.*, 2018 ▸). Conventional small-angle X-ray scattering (SAXS) measurements cannot easily deduce such information as the scattering intensities of the macromolecules, and the associated ions are convoluted into the scattering amplitudes proportionally to their volume and contrast. Using ASAXS and tuning the X-ray energy around an element’s X-ray absorption edge allows for the modulation of the element’s scattering amplitudes, in terms of magnitude as well as phase-shift contributions, to the final scattering profile. With this approach it becomes possible to extract the coherent scattering contributions made by the macromolecules of a sample as well as contributions arising from time and spatially correlated bound ions.

However, the general application of ASAXS is restricted to the atoms having absorption edges within the X-ray energy range accessible on synchrotrons. The most abundant elements in soft matter and biological samples (like carbon, nitro­gen, oxygen, sodium, potassium and calcium) have relatively low energy absorption edges, limiting the practical application of ASAXS for biological macromolecules. Some applications exploit the use of soft X-rays (*E* < 4 keV) at resonant scattering conditions for soft-matter samples (Ingham *et al.*, 2018 ▸; Liu *et al.*, 2016 ▸; Salamończyk *et al.*, 2017 ▸). We note that at low energies resonant scattering is sometimes called RoSXS or ASAXS. These measurements demand high-vacuum conditions and extremely thin samples and are presently not suitable for bioSAXS. The atoms commonly used for ASAXS investigations range from iron to yttrium, where a well resolved *K*-absorption edge is utilized, and also heavier atoms like gold and platinum using their *L* edges. Conveniently, under specific conditions, lighter elements can be isomorphously replaced with heavier elements (*e.g.* calcium by terbium or magnesium by strontium) to obtain more appropriate ASAXS conditions (Dingenouts *et al.*, 2004 ▸; Andresen *et al.*, 2004 ▸; Goerigk *et al.*, 2004 ▸; Austin *et al.*, 1987 ▸; Kilhoffer *et al.*, 1980 ▸; Wallace *et al.*, 1982 ▸), although the integrity of the biomacromolecules, especially the stability of proteins (*e.g.* against aggregation), must be tested upon such substitutions. A further challenge for ASAXS on biological and biologically relevant soft-matter systems is the low volume fraction of these ions compared with the already low volume fraction of the dilute biopolymers with which they associate. As SAXS intensities are proportional to the volume squared, the summed contribution of associated ions and the resulting signals are extremely low. These points result in particular and high instrumental demands compared with hard-matter systems (Barnardo *et al.*, 2009 ▸; Ilavsky *et al.*, 2009 ▸).

For an ideal (monodisperse, non-interacting) macromolecular solution, the SAXS signal can be written as
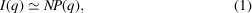
where *N* describes the number density of the particles and *P*(*q*) the particle form factor. Here, the intensity is given as a function of momentum transfer *q*,
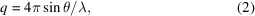
where 2θ is the scattering angle and λ denotes the X-ray wavelength. The form factor, *P*(*q*), describes the overall structure of the particles of volume *V*_P_ and can be expressed as
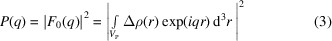
with
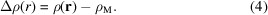
Here *F*_0_(*q*) is the scattering amplitude, ρ(*r*) is the scattering length density distribution of the particles at the position *r* and *ρ*_M_ denotes the (average) solvent scattering length density. The scattering contrast distribution, Δρ(*r*), is a measure of the ‘excess electron density’ of the particles in solution and represents the difference between the particles’ scattering length density and the scattering length density of the solvent.

The description of the scattering amplitude as a coherent superposition of scattered electromagnetic waves from free unbound electrons is a good approximation in the small-scattering-angle limit. It becomes invalid for X-rays with an energy close to the absorption edge of an atom. In this case, the electron-shell configuration of the atom makes a non-ignorable contribution to the scattering process as an electron can be excited from the inner shell. In the course of this, the incoming photon is absorbed by the atom, and the scattering amplitude is decreased. The hole generated at the inner shell is filled by an electron from an upper shell accompanied by emittance of a fluorescence photon.

More formally, the X-ray scattering factor *f*(*E*) of an atom, which describes the scattering amplitude of an element at zero scattering angle, can be written as a complex function having resonant and non-resonant contributions:
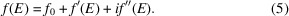
The non-resonant contribution *f*_0_ is given by the total number of electrons of the atom. The energy-dependent real and imaginary parts *f*′ and *f*′′ are related by the Kramers–Kronig relation (Kramers, 1926 ▸; Kronig, 1926 ▸). The relation describes the effect on the amplitude caused by the binding energy of an electron shell and an absorption term (causing a phase lag in the amplitude), respectively. Most importantly, *f*′(*E*) and *f*′′(*E*) only change significantly within a few electronvolts (eV) of the absorption edge of the respective atom, this change being monotonic. Far away from the edge, the overall weak contribution of *f*′(*E*) and *f*′′(*E*) is nearly constant and, thus, their variation can be safely ignored in conventional SAXS measurements.

ASAXS utilizes the measurements in the vicinity of a specific element’s absorption edge, where changes in *f*′(*E*) result in modification of the scattering contrast. In the case of ASAXS from particles in solution, following equation (4), the scattering contrast can be formally written for the X-ray scattering factor of an atom as
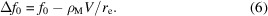
Here, *r*_e_ = 2.818 × 10^−15^ m is the classical electron radius and *V* is the volume of the atom that is inaccessible to a solvent with a scattering length density *ρ*_M_. The total energy-dependent scattering factor of an atom in the solution can therefore be written as
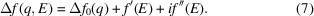
The energy-dependent scattering length density of the particle in the solution can then be written as
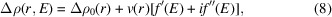
where Δρ_0_(*r*) is the energy-independent scattering length density distribution, *i.e.* the conventional scattering contrast in equation (4). The second term *v*(*r*) denotes the resonant scattering length density distribution, which describes the spatial distribution of anomalous atoms within the particle. The Fourier transformation of Δρ(ρ, *E*) yields the scattering amplitude:
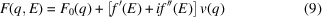
with 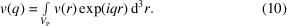
Here, the non-resonant and resonant amplitudes are denoted as *F*_0_(*q*) and *v*(*q*), respectively. Since 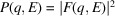, the scattered intensity is expressed as 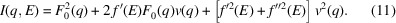
This equation contains three contributions: the non-resonant term 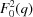, a cross term between the non-resonant and resonant contributions, and the purely resonant term 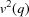 (Stuhrmann, 1985 ▸). The Fourier transform of 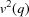 directly provides the spatial distribution of the resonant atoms (Dingenouts *et al.*, 2004 ▸; Goerigk *et al.*, 2004 ▸). In conventional SAXS measurements, the anomalous terms have insignificant contributions. Only by measuring the intensity at different energies across the absorption edge of the resonant atoms where *f*′(*E*) and *f*′′(*E*) show significant changes is it possible to determine the contributions from the resonant and non-resonant parts separately.

This article reports the first ASAXS measurements on biological macromolecules performed at the EMBL P12 bioSAXS beamline at the PETRA III storage ring (DESY, Hamburg, Germany). We describe the instrumental setup along with the experimental and analysis procedures allowing one to reliably extract and analyse anomalous signals from dilute macromolecular solutions. ASAXS studies of the iron-storage protein ferritin and apoferritin hosting cobalt as anomalous atom are presented to illustrate the setup’s potential.

## 2. Material and methods   

### 2.1. Sample preparation   

Bovine serum albumin (BSA) samples were prepared by directly dissolving BSA powder (Sigma; catalogue No. 05470) in 10 ml of 25 m*M* HEPES, 50 m*M* NaCl, 3%(*v*/*v*) glycerol pH 7, to a final concentration of 2.3 mg ml^−1^. Both the protein sample and buffer were 0.22 µm filtered before the SAXS measurements.

Equine spleen apoferritin and iron ferritin were diluted from their supplied stock solutions (Sigma; catalogue Nos. A3660 and F4503, respectively) in 25 m*M* 3-morpholinopropane-1-sulfonic acid (MOPS), 50 m*M* NaCl, 3%(*v*/*v*) glycerol pH 8.5, to approximately 4.2 and 4.5 mg ml^−1^. The concentration of apoferritin was estimated at A280 nm using an *E*_0.1%_ of 0.728. For ferritin, the concentration was calculated from the quoted value of the stock solution from the supplier (125 mg ml^−1^), taking into account the volume of buffer added to the stock (482 µl buffer to 18 µl ferritin) and the final dilution factor. Both samples were dialysed overnight at 277 K against the MOPS/NaCl buffer before being centrifuged at 30 000*g* for 30 min at 277 K immediately prior to the SAXS measurements. Micrometre-filtered post-dialysis MOPS/NaCl buffer was used as the solvent blank.

‘Cobalt-ferritin’, Co-ferritin, was prepared by following the procedure described by Kim *et al.* (2005 ▸) with slight modifications. In detail, the preparation involved taking equine spleen apoferritin and loading the protein with Co^2+^, followed by the oxidation of the Co^2+^ to Co^3+^. Apoferritin from the supplied stock (50% glycerol, 75 m*M* NaCl) was diluted fivefold in 25 m*M* MOPS, 50 m*M* NaCl pH 8.5, to a concentration of 6.4 mg ml^−1^ (assessed at A280 nm). The final sample volume was 1 ml (200 µl apoferritin stock was added to 800 µl of MOPS buffer). Assuming an average molecular weight (MW) of the protein of 480 kDa, the molar concentration of the diluted apoferritin is approximately 13 µ*M*, equating to a total of 13 nmol of apoferritin in the final sample volume. All subsequent steps were performed on ice in sealable plastic Eppendorf tubes. Successive 25 µl aliquots of a freshly prepared 50 m*M* CoSO_4_·0.7H_2_O solution (made in water) were added drop by drop to the apoferritin sample with 3 min incubations on ice between aliquot additions. Cobalt was added to a final molar ratio of 2000 Co^2+^ atoms per mol of apoferritin (a total of 520 µl of the 50 m*M* CoSO_4_·0.7H_2_O were added to the 1 ml protein sample). The Co^2+^/apoferritin mixture was left to incubate on ice for 1.5 h. At this point, to 475 µl of the Co^2+^/apoferritin sample were quickly added 25 µl of freshly prepared 0.3%(*v*/*v*) H_2_O_2_ (in ice-cold water) with vigorous mixing [final H_2_O_2_ = 0.015%(*v*/*v*)]. The Co-apoferritin/H_2_O_2_ mixture was spun at 30 000*g* for 5 min at 277 K and then transferred to room temperature for approximately 3 h; on occasion the sample tube’s lid was briefly opened and closed to relieve pressure. The Co-ferritin was dialysed overnight at 277 K against four successive changes of 25 m*M* MOPS, 50 m*M* NaCl, 3%(*v*/*v*) glycerol pH 8.5. The sample was then centrifuged at 30 000*g* for 30 min, and an aliquot of 0.22 µm-filtered post-dialysis buffer (from the last dialysis exchange) was used as a matched solvent blank for the SAXS measurements. According to the dilution factors through the Co-ferritin preparation protocol, the final protein concentration used for SAXS was estimated at 2.7 mg ml^−1^.

### 2.2. Instrumental setup   

A challenge for ASAXS studies on biological and biologically relevant soft-matter samples is the low volume fraction of anomalous atoms in these samples, leading to extremely weak anomalous signals. Furthermore, caution must be taken to avoid radiation damage of the samples (Schroer *et al.*, 2018 ▸; Jeffries *et al.*, 2015 ▸). The P12 beamline is highly optimized for SAXS experiments on biological and soft-matter samples. The setup uses an in-vacuum capillary to reduce background scattering contributions. Also, an automated and continuous flow operation protocol reduces the effects of radiation damage (Blanchet, Spilotros *et al.*, 2015 ▸). The same capillary is used for the buffer and sample measurements, allowing for reliable background subtraction. The same spot on the capillary is illuminated, reducing ambiguities due to, for example, different wall thicknesses (Round *et al.*, 2015 ▸).

The beamline is equipped with an Si(111) double-crystal monochromator and a focusing mirror system, providing a flux of 5 × 10^12^ photons s^−1^ with a beam size of 120 × 200 µm (vertical × horizontal, FWHM) at the sample position. The energy bandwidth is of the order of Δ*E*/*E* = 0.01% with tuneable energy from 4 to 20 keV. However, energies below 6 keV are challenging to use because of absorption in the downstream optics. Prior to the ASAXS experiments, the monochromator’s energy was calibrated by performing an X-ray absorption scan at the copper *K*α absorption edge of 8980.5 eV. A copper foil was placed into the X-ray beam and the energy was scanned while detecting the transmitted beam with the aid of a diode. We employed a standard Pilatus 2M detector for the measurements.

For ASAXS measurements, it is essential to monitor the incoming and the transmitted intensities of the sample. These are assessed by an intensity monitor before the sample, consisting of a thin zirconium foil which is passed by the X-ray beam and creates scattered photons. The scattered photons are then detected by a diode. The transmitted signal was detected by an active beamstop. The beamstop was tested in previous studies to demonstrate its efficiency and linearity (Blanchet, Hermes *et al.*, 2015 ▸).

### 2.3. Measuring procedure   

The energy cycles for Co and Fe are summarized in Table 1 ▸. To perform reliable measurements and reduce systematic errors, each energy series was measured several times, *e.g.* the series for the sample containing cobalt (absorption edge at 7708 eV) displayed in Table 1 ▸ was measured and then restarted again at the initial energy of 7634 eV. This cycle was repeated at least three times.

The data sets from each cycle at the same energy points were subsequently compared and averaged if no variation in the intensities between these data was observed. Data curves with significant deviations were discarded. The measuring protocol was similar to the one described by Sztucki *et al.* (2010 ▸). At each point during the energy cycle we measured an intensity calibration standard (water) and the buffer. As a further reference, a BSA protein solution containing no anomalous atoms was used to check for possible errors and fluctuations induced by the averaging and normalization at each energy step. The effects of radiation damage or other systematic deviations between the collected data frames (*e.g.* capillary fouling by the sample) were assessed on the fly by an automated software pipeline to ensure that only statistically equivalent sample and buffer frames were used for subsequent data processing and reduction, as described elsewhere (Franke *et al.*, 2015 ▸, 2012 ▸). The *q* axis was measured once at the edge of the sample using silver behenate as angular calibrant and then rescaled on the basis of the known energy shift. The energy threshold of the detector was set to half of the energy value of the edge under consideration.

### 2.4. Calibration and data analysis   

To quantitatively compare ASAXS data, it is necessary to normalize all measured intensities to an absolute scale (cm^−1^). This normalization was based on the scattering from the intensity calibration standard (water). As data calibration is essential for reliable ASAXS investigations and even more for samples with low anomalous signals, we also decided to measure a further standard (here, BSA) to check that all normalization and calibration steps worked adequately.

The normalization, energy recalibration and averaging were done with MATLAB scripts. For the separation of the anomalous signal, a quadratic method proposed by Ballauff & Jusufi (2006 ▸) was implemented in a MATLAB script. We followed the approach proposed by Ballauf and Jusufi, neglecting the contribution of *f*′′, which is valid for energies below the edge. In short, the scattered intensity at a given *q* value is plotted as a function of the energy and a quadratic polynomial is fitted to the curve: 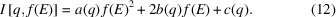
The amplitude of the quadratic contribution *a*(*q*) yields the resonant part; the non-resonant part is given by the static offset *c*(*q*), and a linear contribution is given by *b*(*q*), which is a cross term including resonant and non-resonant parts. Having these terms available, it is possible to determine the number of excess atoms *n* by using the equation 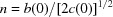 (Pabit *et al.*, 2010 ▸). For the determination of the *p*(*r*) function the program *GNOM* from the *ATSAS* suite was used (Manalastas-Cantos *et al.*, 2021 ▸; Franke *et al.*, 2017 ▸; Svergun, 1992 ▸). *p*(*r*) is an indirect Fourier transformation yielding the distribution of distances between volume elements weighted by the excess density distribution and can be calculated as described by Svergun & Koch (2003 ▸):
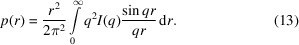
For the cobalt-containing samples’ data, a model fitting was performed using the program *SASView* (https://www.sasview.org), where all curves measured for the sample were fitted simultaneously. The model consisted of a spherical model with a core and two additional shells. Here, the fitting parameters were restrained to obtain a stable fit and reasonable physical model. Parameters like the scattering length density (SLD) of the outer shell, the shell thickness and the core diameter were coupled between the data sets from the different energies. The SLD of the core was fixed to the SLD of the solvent. Only the scattering length density of the inner shell was allowed to vary for each energy to account for the anomalous signal change. This modelling was only performed for Co-containing samples; the ferritin data did not allow for such an approach because the scattering length of the protein shell was too low (see Section 3). Furthermore, *ab initio* shape calculations were performed by running *DAMMIN* (Svergun, 1999 ▸) on the scattering intensity after it had been manually processed by *GNOM* (Franke *et al.*, 2017 ▸).

## 3. Results and discussion   

**Figure 1:**
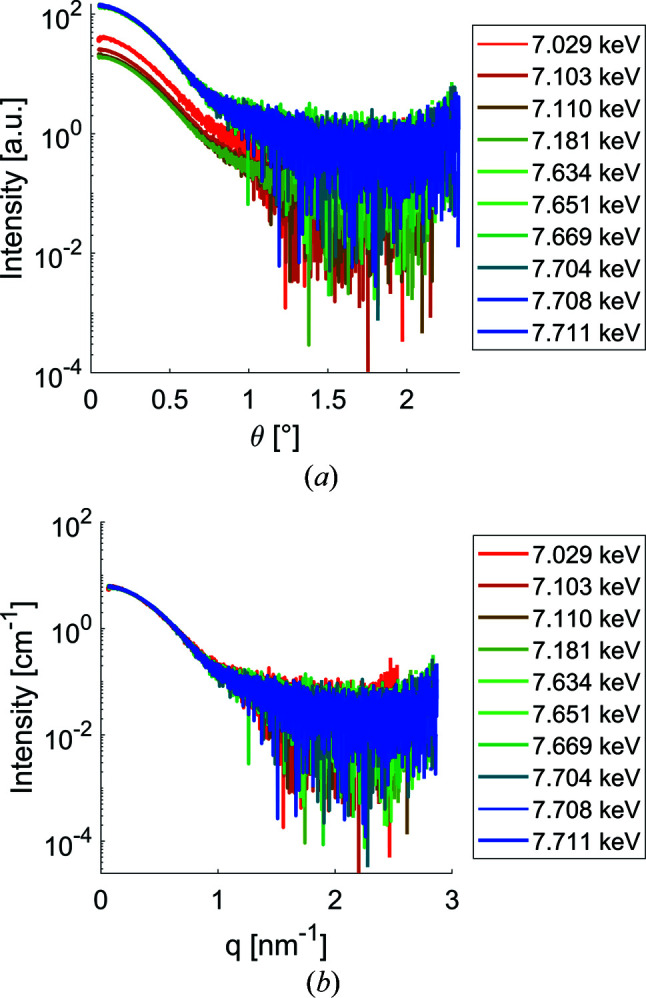
BSA reference measurements at different energies around the iron and cobalt edges. The BSA data were collected at each energy cycle before the respective samples were measured. (*a*) Data provided by the P12 data reduction pipeline (intensity as a function of the scattering angle). (*b*) The data normalized to absolute scattering intensities and converted into the appropriate *q* space.

Fig. 1 ▸ shows SAXS data measured from the BSA samples at different X-ray energies for the ASAXS measurements across the Co and Fe absorption edges. The BSA data were measured as a reference sample to check that the calibration and normalization are performed correctly. Owing to the extremely low anomalous signal from biological macromolecules, data calibration is of major importance for reliable extraction of the relevant scattering features. The data from BSA (which does not contain anomalously scattering atoms in the studied energy range) are shifted mainly vertically as a result of the changed photon flux. Also, a slight shift in the scattering angle grid can be seen as a consequence of the changing wavelength (a change in the energy of 100 eV leads to a shift on the angular axis by about nine data points for the given detector setting). After normalization of the incoming flux with respect to water and transformation of the angular axis to the *q* scale, the data overlap perfectly, demonstrating the reliability of the normalization procedures and the stability of the P12 setup [see Fig. 1 ▸(*b*)].

**Figure 2:**
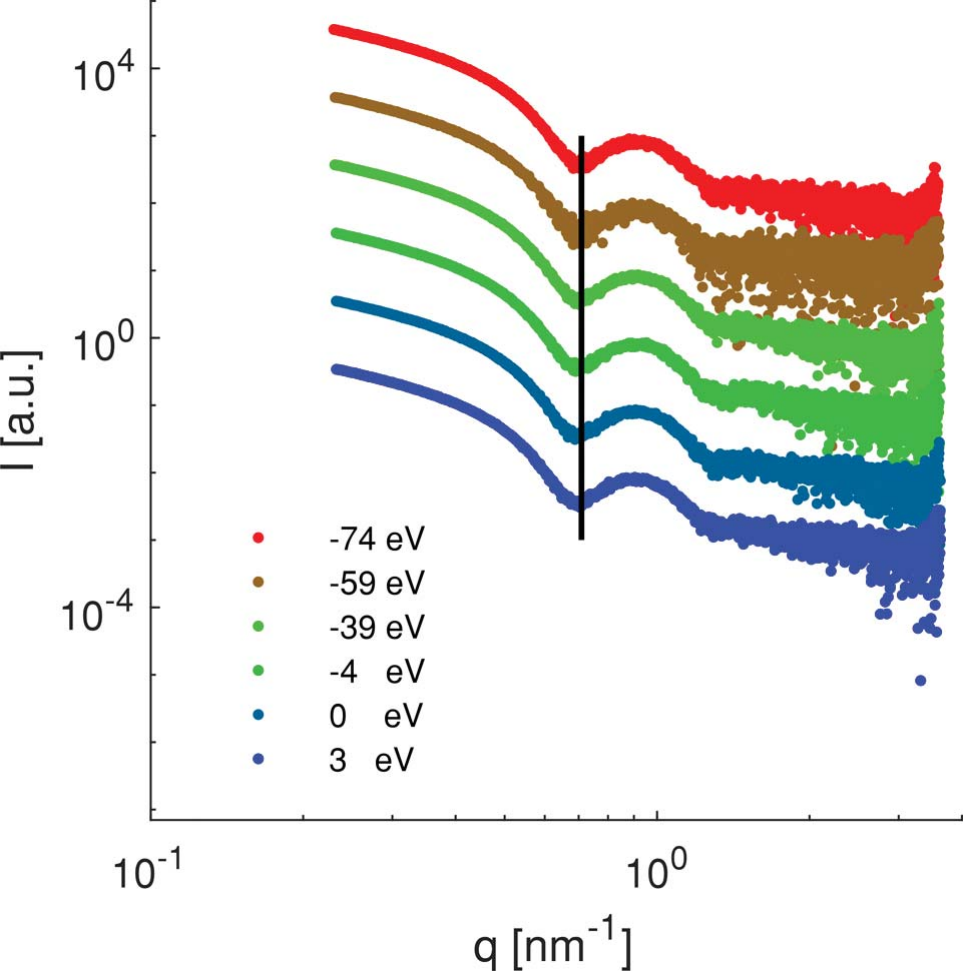
ASAXS curves of apoferritin loaded with cobalt. Measurements were done at different energies around the Co *K* edge of 7.708 keV. The curves are shifted vertically for clarity. The black line is a visual guide drawn at the position of the minimum for the photon energy of 7.624 keV.

We have performed measurements on solutions of ferritin and apoferritin loaded with cobalt. For the preliminary characterization, apoferritin was measured. These data are provided in the supporting information (SI) Fig. S1. The data display no signs of aggregation. Fig. 2 ▸ shows the set of scattering curves for the cobalt-containing apoferritin (vertically shifted for clarity). A comparison of the scattering data from apoferritin loaded with cobalt and pure apoferritin is given in Fig. S1. ASAXS measurements were performed at five energies around the Co edge at 7.708 keV (four below and one above the edge). We note that, by performing an intensity scan as a function of the energy across the Co edge of the apoferritin/cobalt sample, no absorption edge could be determined as the cobalt concentration was too low. Also, the differences in the curves at the used photon energies are only minimal. However, a small shift (Δ*q* = 0.031 nm^−1^) of the first minimum can be observed; this minimum shifts to lower *q* values as the energy is increased towards the adsorption edge (solid line in Fig. 2 ▸). The shift due to an improper energy calibration would have been much smaller (0.008 nm^−1^), and this comparison indicates that the shift is indeed due to the resonant effect. The change of the integrated scattered intensity is about 1.5% when varying the energy by 74 eV (see Fig. S2 in the SI). These small changes are close to the effect expected and highlight the low background contribution at P12, which is necessary to perform precise experiments to detect such an effect. Independently for all curves, the radius of gyration was determined (see Table 2 ▸).

The radius of gyration shows no systematic variation at the different energies. However, the samples showed a high degree of aggregation, resulting from the apoferritin’s loading with cobalt. As this contribution to the scattering curve is well separated from that of the Co-loaded apoferritin monomer, the data were truncated at a lower *q* value of 0.3 nm^−1^ for further analysis. This truncation at very small angles does not hamper the extraction of the structural parameters of monomeric apoferritin. Given the specific shape of apoferritin as a hollow sphere, further structural information is contained in the *q* range larger than 0.3 nm^−1^, and in the first minimum for which the contribution of larger aggregates is absent.

**Figure 3:**
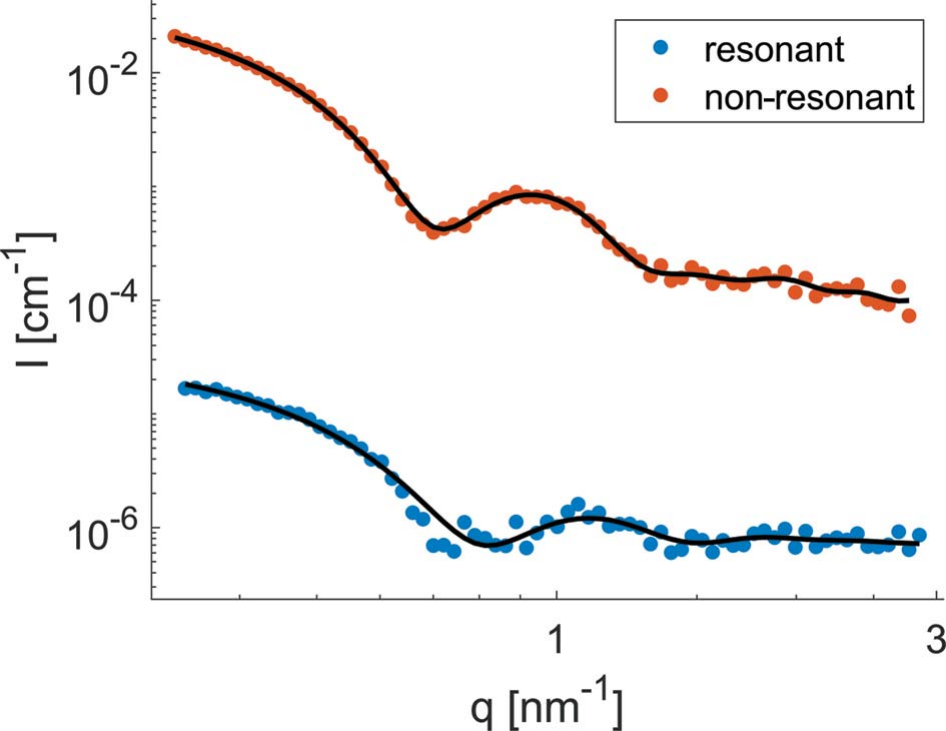
Resonant *v* ^2^(*q*) and non-resonant 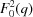 curves of apoferritin loaded with cobalt. The black lines show the fit computed from the *p*(*r*) function restored by *GNOM*.

For quantitative analysis, a decomposition of the resonant and non-resonant parts was done (Sztucki *et al.*, 2012 ▸; Ballauff & Jusufi, 2006 ▸), for which all curves below the edge were used. Fig. 3 ▸ shows the extracted resonant signal *v* ^2^(*q*) along with the non-resonant contribution 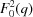. The two curves exhibit different shapes and distinct features. The resonant curve shows a first minimum at *q* = 0.78 nm^−1^ while *F* ^2^(*q*) has a minimum at *q* = 0.72 nm^−1^. Furthermore, the lower polydispersity of the resonant part is evident because the minimum is more clearly visible than that of the non-resonant data. The radii of gyration calculated from the resonant and non-resonant terms are 6.10 ± 0.07 nm and 5.99 ± 0.07 nm.

**Figure 4:**
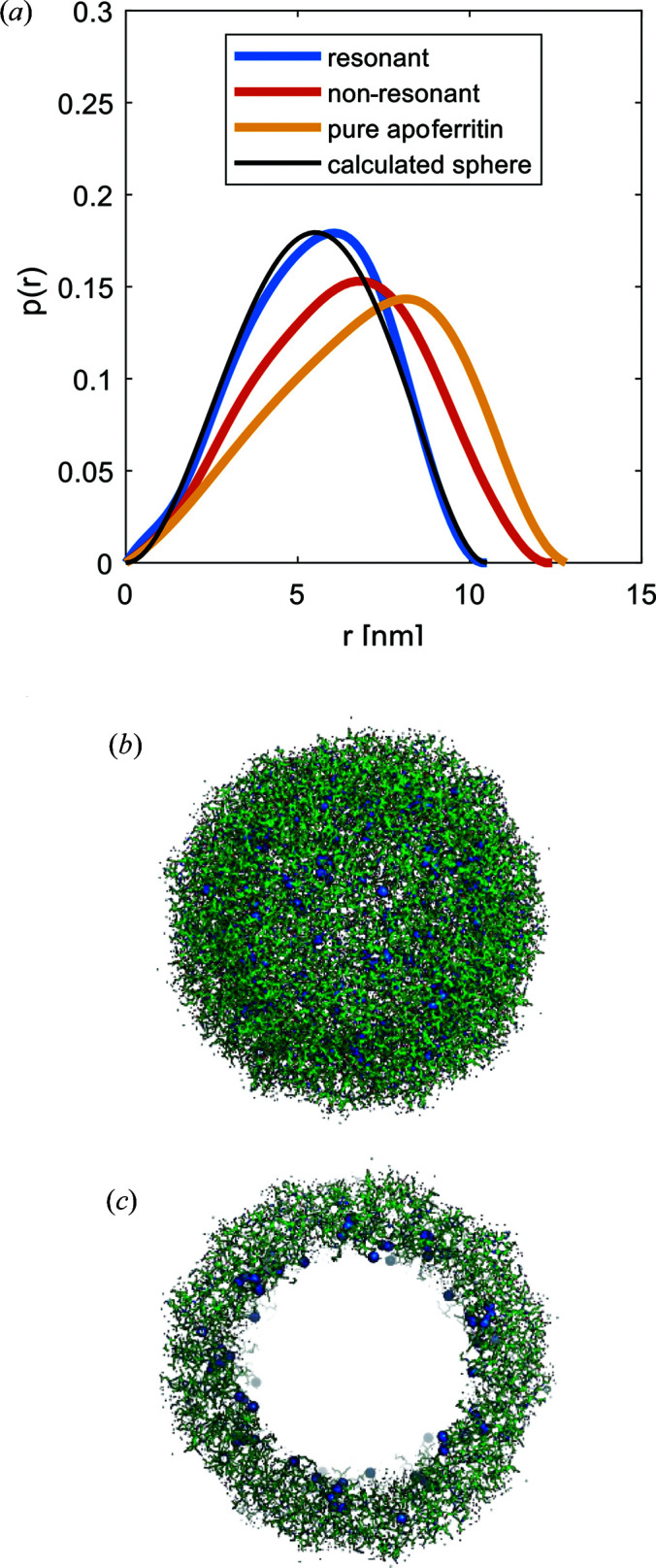
(*a*) Normalized *p*(*r*) function of apoferritin along with the *p*(*r*) function calculated from the extracted resonant and non-resonant parts of the loaded apoferritin sample. The fits to the data to obtain *p*(*r*) are shown in the SI. For comparison, *p*(*r*) for a sphere is plotted. Additionally, *p*(*r*) for the unloaded apoferritin is plotted, displaying a shape typical for a hollow sphere. (*b*) Crystal structure of Co(II) wild-type frog M-ferritin (PDB code 3ka4). (*c*) Cut through of the structure of Co(II) wild-type frog M-ferritin. Bold beads represent Co atoms forming a fuzzy inner shell within the ferritin core.

We further calculated the *p*(*r*) functions for the decomposed data set. The fits are displayed in Fig. 3 ▸, and the results are shown in Fig. 4 ▸(*a*). The shape extracted from the non-resonant part of Co-loaded apoferritin is similar to that of the unloaded apoferritin. Both curves exhibit an asymmetric shape with the maximum shifted to larger distances *r*, which is characteristic for core–shell particles, in contrast to the sphere’s symmetric bell-shaped curve. For the Co-loaded sample, this shift of the maximum position is less pronounced than for unloaded apoferritin, indicating a different density distribution. For the resonant part *v* ^2^, the asymmetry of the curve is less prominent but still visibly distinct from the sphere’s *p*(*r*) function. The *p*(*r*) functions of the resonant and non-resonant parts exhibit an overall asymmetric shape but with a different maximum extension [Fig. 4 ▸(*a*)]. As the resonant part is only sensitive to the Co atoms, this indicates cobalt accumulation within a smaller volume similar to a hollow sphere, *i.e.* at the protein’s inner surface. The curves fitted to the *p*(*r*) functions allow us to estimate the intensity at zero scattering angle and, thus, to estimate the number of excess ions *n* to be 357.

An additional analysis was done by fitting all measured curves at the five energies simultaneously using the program package *SASView*. The details are described in Section 2, and Table 3 ▸ summarizes the values obtained by the fitting.

The model yields an outer diameter of 11.4 nm, with a core diameter of 3.4 nm and a thickness of the inner and outer shells of 2.2 and 1.7 nm. The SLD obtained by fitting the inner shell as a function of energy is summarized in Table 3 ▸. The fits to the data are shown in Fig. S4. The SLD of the inner shell containing the cobalt atoms decreases from (9.00 ± 0.01) × 10^−4^ nm^−2^ to (8.67 ± 0.01) × 10^−4^ nm^−2^ with the change in energy as expected. The SLD for the inner part is lower than that of the protein shell, indicating that the cobalt density in the inner part is low and no dense cobalt layer is formed.

Thus, the cobalt atoms are located in the inner core of apoferritin, filling the protein’s inner cavity. This result agrees with other publications where apoferritin is used as a reactor for nanoparticles and with the models of the Co(II) wild-type frog M-ferritin (PDB code 3ka4) displayed in Figs. 4 ▸(*b*) and 4 ▸(*c*) (Gálvez *et al.*, 2008 ▸; Tosha *et al.*, 2010 ▸).

In conclusion, the ASAXS data show that apoferritin’s outer diameter is reduced upon cobalt loading, and the protein adopts a more compact shape. Cobalt itself accumulates at the inner surface of apoferritin within a layer thickness of 2.2 nm, as indicated by the *p*(*r*) from the decomposed curves and the independent data fitting procedures.

**Figure 5:**
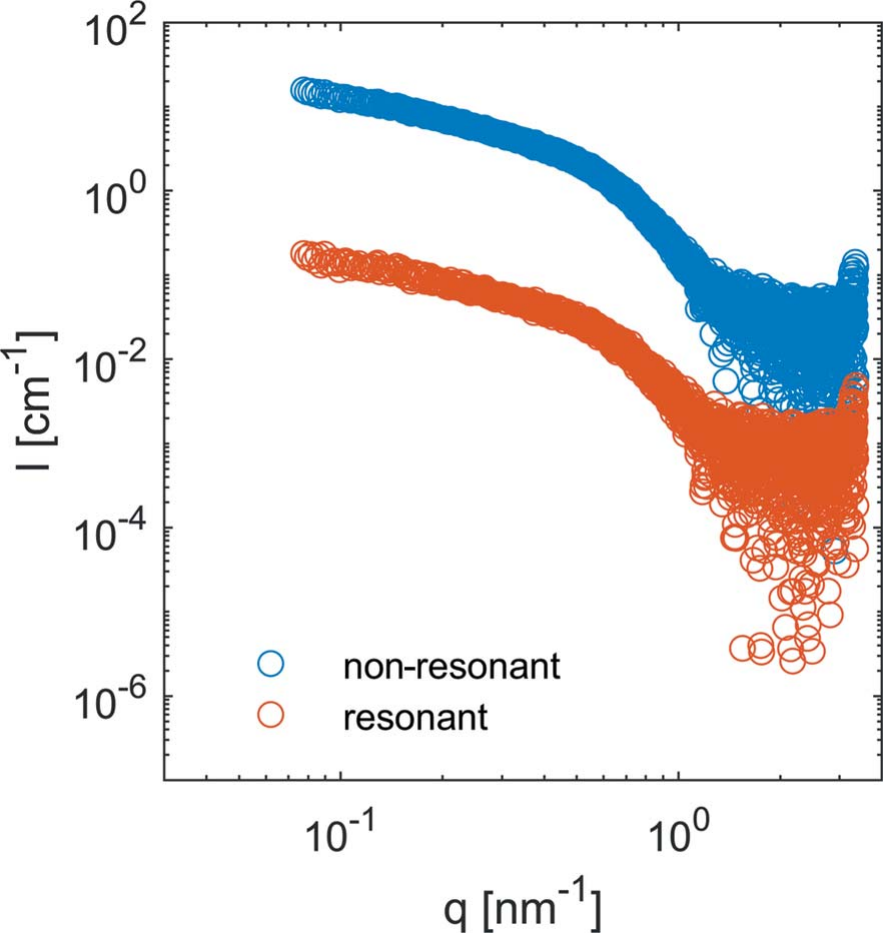
The resonant and non-resonant terms for ferritin calculated using equation (10).

**Figure 6:**
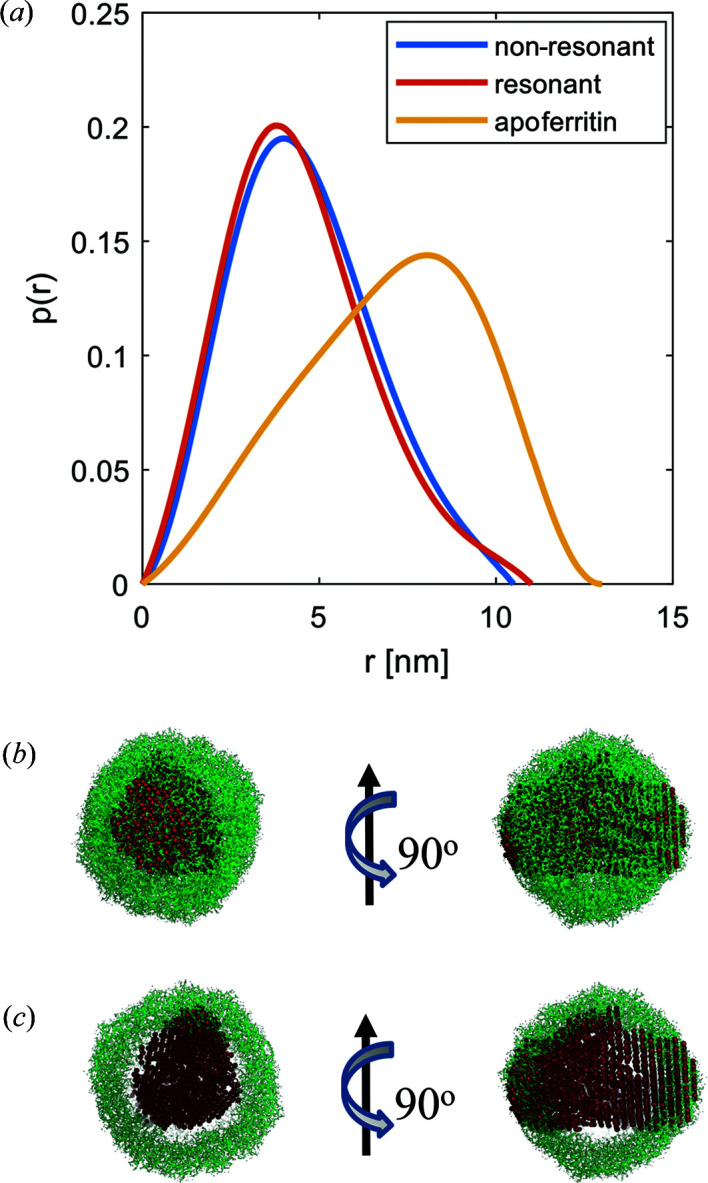
(*a*) Normalized *p*(*r*) curves calculated for resonant and non-resonant scattering contributions of ferritin. As a reference, *p*(*r*) from apoferritin is shown (scaled down by a factor of 1500). (*b*) *Ab initio* model of measured ferritin aligned with apoferritin (PDB code 3f32; Vedula *et al.*, 2009 ▸). High-contrast inner core packed with iron atoms. (*c*) A cut through the model in panel (*b*).

Further experiments were performed on ferritin at the iron *K* edge (7.110 keV). The collected ASAXS curves are presented in Fig. S5, and the extracted resonant and non-resonant terms are displayed in Fig. 5 ▸. The extraction of the resonant and non-resonant curves was achieved by using the SAXS curves from all energy points. The radii of gyration calculated from the resonant and non-resonant terms are 3.81 ± 0.05 nm and 3.91 ± 0.05 nm, respectively, indicating a small yet significant difference. The ratio of the non-resonant to resonant extracted intensity as a function of *q* is shown in the Fig. S7. The *p*(*r*) functions calculated from the two terms are displayed in Fig. 6 ▸(*a*) along with the reference *p*(*r*) from apoferritin.

The *p*(*r*) functions are bell-shaped curves with a maximum size of 11 nm, distinctly differing from apoferritin. Also, relatively small but systematic differences are observed between the resonant and non-resonant curves of ferritin. To understand this behaviour, we compare the non-resonant atomic scattering factors of carbon/oxygen (6.025/8.006) with that of iron (22.03), *i.e.* the non-resonant scattering length of iron is about three times larger than that of the other atomic species. Given a much higher iron content in the inner part of ferritin, the scattering from the iron-rich part significantly exceeds that from the weakly scattering protein shell. Therefore, the recorded scattering is mainly reflecting the inner core of ferritin with densely packed iron atoms, while the outer shell gives a minor contribution. The curves fitted to the *p*(*r*) functions also allow us to estimate the intensity at zero scattering angle and, thus, the presence of 1300 excess iron ions in the core.

Figs. 6 ▸(*b*) and 6 ▸(*c*) provide overlays of an *ab initio* model calculated from the measured ferritin curve with the atomic structure (the *DAMMIN* fits are presented in Fig. S6). This comparison further supports the notion that the measured ferritin core is highly loaded with iron and this core is primarily ‘seen’ by X-rays owing to the high scattering contrast (Kim *et al.*, 2011 ▸; Pan *et al.*, 2009 ▸). The protein shell only induces small differences between the resonant and non-resonant parts.

## 4. Conclusion   

The presented ASAXS analysis demonstrates the incorporation of cobalt into the inner core of apoferritin, the cobalt moiety forming a structure similar to that of a hollow sphere. For this test system, ASAXS unambiguously shows that cobalt is located in the protein’s inner part. Using conventional SAXS, the determination of the cobalt portion would have been impossible because the cobalt fraction and protein moiety have similar SLDs. In contrast, the ASAXS study at the iron absorption edge demonstrates that the iron atoms do not form a hollow sphere but occupy the entire particle core, forming a compact structure. Given the large number of iron atoms in the apoferritin, ASAXS analysis allows one to determine even the small differences in the iron-atom content between the protein shell and its core.

These experiments demonstrate the bioSAXS P12 beamline capabilities for anomalous scattering experiments on soft matter and protein solutions. Thanks to the combination of the high flux and low background, interpretable ASAXS data can be collected even on samples with relatively small quantities of anomalous atoms, significantly enriching the structural information in the scattering data.

## Supplementary Material

Supporting figures. DOI: 10.1107/S1600576721003034/ge5089sup1.pdf

## Figures and Tables

**Table 1:** The energies at which measurements for the Co and Fe samples were performed

Energy point	Co (eV)	Fe (eV)
1	7634	7029
2	7651	7103
3	7669	7110
4	7704	7181
5	7708	–
6	7711	–

**Table 2:** Radius of gyration, *R*_g_, of apoferritin loaded with cobalt measured at different photon energies As a reference, *R*_g_ of the unloaded apoferritin was determined to be 5.9 nm.

Energy (eV)	*R*_g_ (nm)	*I*_0_ (cm^−1^)
7634 (Δ*E* = −74)	5.5 ± 0.1	0.57 ± 0.01
7651 (Δ*E* = −59)	5.4 ± 0.1	0.57 ± 0.01
7669 (Δ*E* = −39)	5.5 ± 0.1	0.58 ± 0.01
7704 (Δ*E* = −4)	5.5 ± 0.1	0.57 ± 0.01
7708 (Δ*E* = 0)	5.5 ± 0.1	0.56 ± 0.01

**Table 3:** Fitting parameters of apoferritin loaded with cobalt to a core–shell system using *SASView*

Energy (eV)	7634 (Δ*E* = −74)	7651 (Δ*E* = −59)	7669 (Δ*E* = −39)	7704 (Δ*E* = −4)	7708 (Δ*E* = 0)
SLD solvent (10^−4^ nm^−2^)	6.39 ± 0.01				
SLD shell (10^−4^ nm^−2^)	1.04 ± 0.01				
Shell thickness (nm)	1.79 ± 0.01				
SLD shell 2 (10^−4^ nm^−2^)	8.67	8.70	8.80	8.89	9.00
Thickness shell (nm)	2.20 ± 0.01				
Core radius (nm)	1.72 ± 0.01				
