# Supplementary material for: ASAXS measurements on ferritin and apoferritin at the bioSAXS beamline P12 (PETRA III, DESY)
Source: J Appl Crystallogr. 2021 May 25;54(Pt 3):830–8. doi: 10.1107/S1600576721003034 (PMC8202030; doi:10.1107/S1600576721003034)
Supplement: Supplementary file 1 [file j-54-00830-sup1.pdf]

JOURNAL OF
APPLIED
CRYSTALLOGRAPHY

Volume 54 (2021)

Supporting information for article:

ASAXS measurements on ferritin and apoferritin at the bioSAXS beamline P12 (PETRA III, DESY)

D. C. F. Wieland, M. A. Schroer, A. Yu. Gruzinov, C. E. Blanchet, C. M. Jeffries and D. I. Svergun

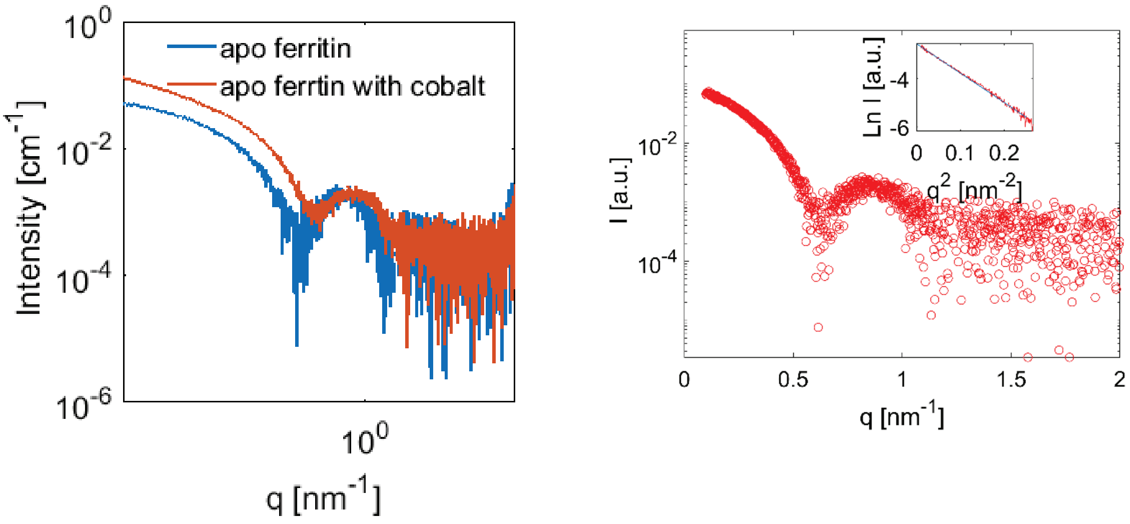

Figure S1 Left: SAXS data of apoferritin and ferritin loaded with cobalt measured at 6keV. Right: pure apo ferrtin including the low q-regime. The inset shows the gunier plot of the low q-regime.

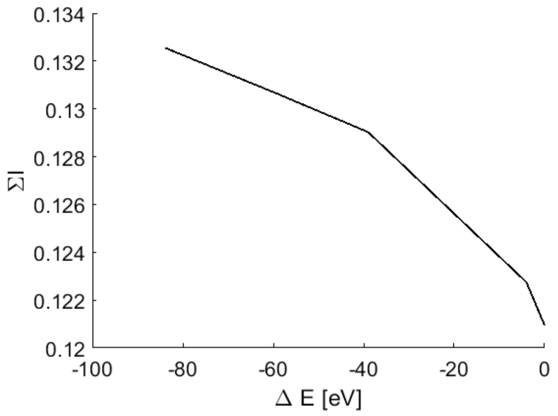

Figure S2 Integrated scattering intensity from 0.3 to 0.35 q [nm^{-1}] of the apoferritin loaded with cobalt as function of the energy difference with respect to the Co absorption edge.

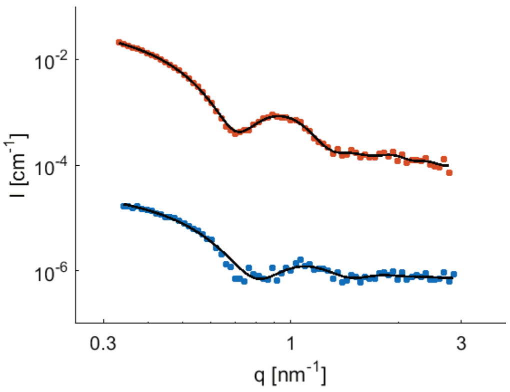

Figure S3 Fits for the P(r) function for apoferritin loaded with cobalt. Blue curve: resonant part; red curve: non-resonant part.

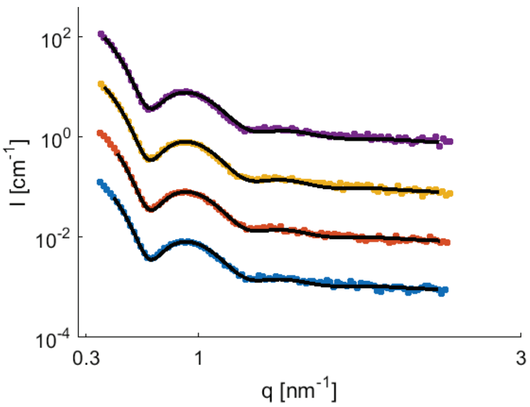

Figure S4 Fits of apoferritin loaded with cobalt to a core shell system (two shells accounting for protein and cobalt and aqueous core) using SASView.

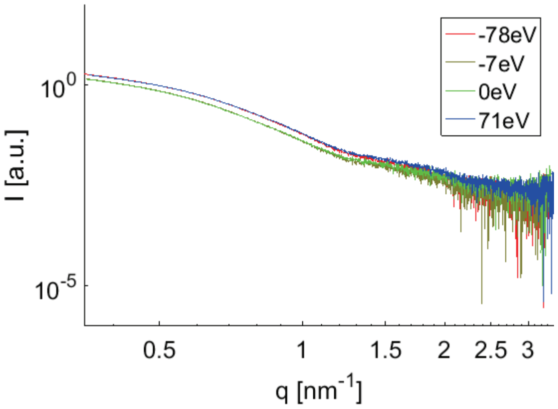

Figure S5 ASAXS curves of ferritin measured at 4 different energies around the absorption edge of 7.110keV. The curves are named by the energy difference to the edge.

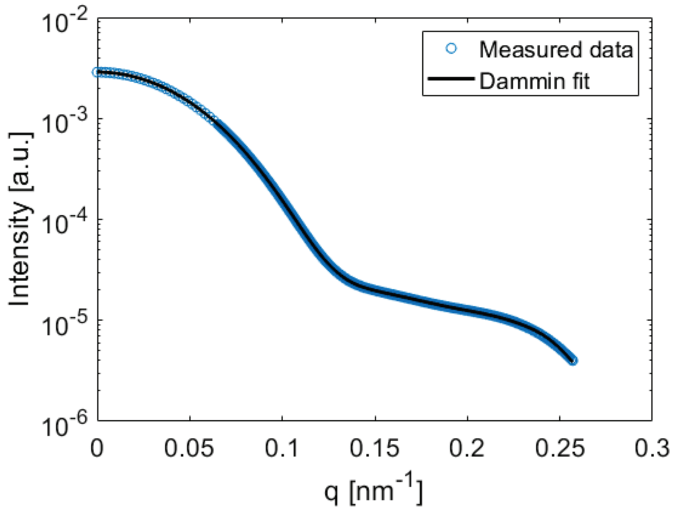

Figure S6 Dammin fits of apoferrtin to describe the structure of the measured sample by a dummy bead model

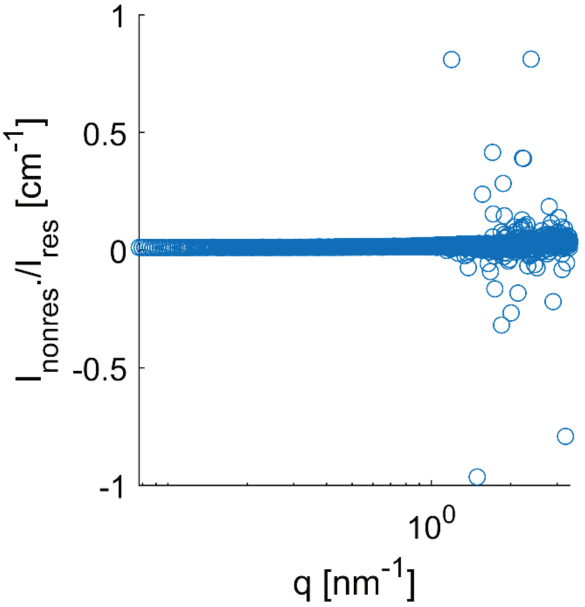

Figure S7 Ratio of non resosnant to the resonsnat term of the ferrtin data.
